# Mental health problems, interpersonal trust, and socio-cultural correlates of corruption perception in Ghana

**DOI:** 10.3389/fpubh.2024.1269579

**Published:** 2024-02-28

**Authors:** Frederick Anyan, Johnny Andoh-Arthur, Stephen Baffour Adjei, Charity Sylvia Akotia

**Affiliations:** ^1^Department of Psychology, Norwegian University of Science and Technology, Trondheim, Norway; ^2^Department of Psychology, University of Ghana, Accra, Greater Accra, Ghana; ^3^Department of Interdisciplinary Studies, Akenten Appiah-Menka University of Skills Training and Entrepreneurial Development, Kumasi, Ghana

**Keywords:** corruption, mental health, ghana, network analysis, corruption perception

## Abstract

**Introduction:**

This study examines the overall levels and effects of corruption perception on mental health while controlling for the effect of interpersonal trust as a routine covariate in studies of corruption.

**Methods:**

Participants (*N* = 730; 60.8% Men; Mean age = 22.13, *SD* = 3.66) were invited to answer a cross-sectional survey. Group mean difference tests and network analysis were performed.

**Results:**

Women, urban dwellers, and those who showed moderate religiosity, and lower nationality reported the highest levels of corruption perception, but the effect on mental health problems was stronger for higher religiosity. The perception that politicians and government officials are corrupt emerged as the most influential to link other corruption perceptions (e.g., state institutions are corrupt). Witnessing corruption among state institutions and government officials and the perception that the rich in society can influence any state institutions and actors showed the strongest and broadest links to depression and anxiety symptoms.

**Discussion:**

The findings suggest that there may be substantial effect of corruption on mental health problems than trust in interpersonal relationships. The relatively high poverty rate in Ghana may explain why those who do not have the financial means or personal connections to meet the demands of bribery and corruption experience a sense of helplessness associated with mental health problems when they perceive that the rich in society can influence state institutions and actors for personal gains. Furthermore, the tendency to remain silent to protect others from being exposed in corruption in order to maintain relationships, or to expose them to ruin relationships, or conform to a culture of corruption either in solidarity or fear of victimisation, may create a psychological burden that may be associated with mental health problems. The implications for reconceptualising corruption as a key social determinant of public mental health are discussed.

## Introduction

Corruption is widespread, especially in developing countries, and it frequently appears in popular and academic debates (1–3). Regional average score of Corruption Perception Index (CPI) in sub-Saharan Africa received the worst ranking, indicating how corrupt the public sector across the region is perceived to be (4). Corruption includes all forms of irregular, unethical, immoral and/or illegal practices and transactions occurring in the process of handling public transactions or in the performance of state/official duties (5, 6). Corruption has been generically defined as the private wealth seeking behaviour of someone who represent the state or public authority, which could be in the form of political, administrative, economic, or petty corruption (7). Lessig (8) offers a definition of institutional corruption that highlights systemic and strategic influence that undermines an institutions effectiveness. According to Lessig, such influences may be “legal, or currently ethical” (8) (pp. 553), but corruption is manifested when (1) the institution’s purpose is diverted, and (2) its ability to achieve its purpose is weakened, leading to diminished public trust in the institution or the institutions inherent trustworthiness.

In the context of a democratic society, every kind of corruption is detested and unwanted as it has far-reaching negative consequences on the economic life and well-being of the population (9). The economic consequence of corruption is well understood and documented in the literature (10). However, not much has been done to explore the mental health consequences and the dangers corruption poses to interpersonal relationships through the erosion of trust. Directly observing corruption or corrupt activities is almost always impossible. Therefore we chose to focus on corruption perception rather than objective corruption or actual corrupt activities in this study by using self-reported instruments to ask participants’ beliefs and impressions of corruption. Additionally, we focus on the negative impacts that different aspects of corruption perception may have on mental health problems measured as suicide risk, anxiety, and depression symptoms in a low-income country of Ghana. We also examined the effect of corruption perception on interpersonal trust, which is a popular covariate in studies that examine attitudes and corruption-related behaviours.

### Effects of corruption on mental health problems

The vast majority of studies examining the effects of corruption have devoted relatively little attention to developing a coherent theoretical framework for explaining the relationship between corruption and mental health (11). Gillanders (12) investigated the mental health cost of corruption using microeconomic data from the Afrobarometer surveys (as evidence from sub-Saharan Africa) and concluded that corruption is a social stressor, which affects the mental health of victims. Van Deurzen (11) followed up the findings by Gillanders (12) in a study which examined corruption as a contextual source of mental illness using data from the 2006, 2012 and 2014 European Social Survey, which involved 99,159 individuals across 24 European countries. Contrary to the ‘social stressor’ view, Van Deurzen (11) argued that loss of income and decline in trust appear to be the main mechanisms through which corruption affects mental health. Bribe payments from victim’s portions of income can worsen their economic situations, and the uncertainties and arbitrariness in extortions can induce helplessness, which are all associated with anxiety and depression (3). Disenfranchising deserving victims from access to healthcare, education, and public administration on the whims of a bribe-seeker who is a corrupt government official, and procedural unfairness and injustice can trigger feelings of anger, hostility, and frustration that may elevate symptoms of anxiety and depression (3, 11). Furthermore, corruption can affect mental health through increases in the salience of corruption or corrupt scandals that are constantly reported via mass media. Shared perceptions of high societal corruption can induce heightened expectations that individuals or their loved ones could become victims (11). In addition, the trust of the citizenry in the local governance and accountability structures of a state becomes seriously eroded due to corruption perception (3). These findings raise the question of whether corruption itself or the perception of corruption have any direct effects on public mental health or other factors mediate the association with mental health problems.

Despite theoretical advances in developing a framework to explain the relationship between corruption and mental health problems, there remains very important questions. For instance, do both the perception of corruption and the participation in corruption exert the same effect on mental health? And what aspect of corruption perception or participation exerts the greatest impact and on what kind of mental health problems? In one study, bribe payments to obtain documents or permits to avoid the police, and the bribe payments to get medical care emerged as the aspects of corruption with the most damaging effect on mental health (12). Additional questions may include, does witnessing corruption exert more impact on mental health than participation in corruption? And does the perception that wealthier people can influence state institutions or government officials for personal gains exert more impact on mental health than the perception that state institutions are corrupt? To respond to these questions, this study investigated four aspects of corruption perception: (i) *the perception that state institutions are corrupt*, (ii) *the perception that politicians and government officials are corrupt,* (ii) *the perception that the rich in society can use their resource for personal gains*, and (iv) *witnessing corruption among state institutions or officials*. These four aspects are investigated in relation to differences in various background characteristics (age and gender) and sociocultural variables (religiosity, nationality, and place—i.e., where one has lived the most in life) as well as their relationships with measures of mental health problems in a novel approach using psychological network analysis.

### Effects of corruption among different social groups

Different groups of people perceive and experience corruption differently and thus, the perception of corruption may depend on how a society, or a group of people understands what constitutes a deviation. Accordingly, the effect of corruption itself or corruption perception on mental health is expected to vary for different social groups. The link between gender and corruption has received some attention (1). For example, Transparency International recently reported that women are constantly exposed to bribery and sextortion, which has become common in many sectors in sub-Saharan Africa, including sectors such as education, police, refugee camps, judiciary, and civil services (4, 13). Sexual harassment and the constant exposure to inappropriate sexual demands can induce helplessness or powerlessness, which can negatively affect the mental health of individuals, especially women who are the more vulnerable victims. Consequently, women may more strongly perceive that either themselves or their loved ones could be unfairly targeted for corruption. Unsurprisingly, a recent study employing a large sample of mostly low-and-middle income countries (LMIC), covering more than 100 countries, reported that female entrepreneurs reported high corruption experiences and perceptions relative to their male counterparts (14). Within the local context of Ghana, it has also been argued that women may not prove any less corrupt than men when exposed to corruptible public sector environments. This was based on a list of research undertaken on the gender–corruption nexus, focusing on the Ghana Police Service (GPS) and the Ghana Education Service (GES) (15). Taken together, we hypothesised that:

*Hypothesis* 1: Women will report higher levels of corruption perception and stronger relationship between corruption perception and mental health problems.

There is a dearth of literature on the relationships between age and corruption and the debilitating effects corruption may have on mental health in sub-Saharan Africa. In a survey of more than 47,000 citizens from 35 countries across Africa in the Global Corruption Barometer (GCB) by the Transparency International (16), it was found that young people aged 18–34 years were more likely to pay bribes than older people aged over 55 years. Elsewhere, using data from Belgium, Denmark, France, Great Britain, Ireland, Italy, Netherlands and Spain in the World Values Survey and the European Values Survey, Torgler and Valev (17) examined how age and cohort effects affect attitudes towards corruption. The authors found that higher age is correlated with lower justifiability of corruption, which means the older a person is the less likely he/she is to justify corruption. These results may not be directly comparable as the former relates to participation in corruption across African countries while the latter relates to attitudes towards corruption in non-African countries. Still, we can find a link between corruption perception and participation such that a person may entertain favourable attitudes to corruption to be able to participate in corruption and therefore, to that extent, the results may be interesting for comparison. As such, we may draw from these results and conclude that, barring contextual and cultural dissimilarities, younger people may score higher on corruption experience or perceptions than older people. This idea seem promising as older people have acquired greater social, political, or economic status, and thus, their stakes mean that the threat or consequence of corruption and the sanctions for corruption would be more costly than younger people (18). We thus hypothesised that:

*Hypothesis* 2: Young adults will report higher levels of corruption perception and stronger relationship between corruption perception and mental health problems.

Religion can influence human social behaviours and actions. The constant exposure to religious content serves to remind religious individuals about their obligation to moral, ethical, and legal conduct or transactions based on religious archetypes in their belief system (19), and encourages the thoughts about supernatural watchers of ethical or unethical behaviours (20). Many researchers have offered different explanations for the link between religiosity and corruption. For example, Sommer, Bloom, and Arikan (21) have argued that it is not just an individual’s level of religiosity that determines whether they will behave in a moral or ethical manner but also, the presence and the salience of religious cues within the individual’s environment potentially spur the moral standards associated with religiosity. As a result, the presence and salience of religion in state institutions should reduce corruption (21). Additionally, the norms and values that are embraced by religious persons go against the use of lies, dishonesty, and deception that feature prominently in corruption (11). Interestingly, the results from the study by Van Deurzen (11) supported the hypothesis that religious people experience stronger effect of corruption on their mental health. Based on this, we hypothesised that:

*Hypothesis* 3: People who score higher on religiosity will report higher levels of corruption perception and stronger relationship between corruption perception and mental health problems.

The question of whether individuals scoring higher on nationality would score higher on corruption perception or participation and report stronger or weaker effects of corruption on their mental health than those scoring lower appears non-existent in the literature. Nationality is not a clearly defined phenomenon (22) and for this reason, some authors have pointed out the futile attempts to objectively define it as well as the difficulty in agreements for a formal definition (23, 24). In this study, we construed nationality to mean membership of a country by a person, encompassing being proud of one’s country with readiness to correct deficiencies, including avoiding or preventing corruption. Based on our definition of nationality, and due to the endemic corruption in Ghana, we reasoned that nationality pride may influence people to conceal the negative experiences and vices in their country, and this may not show a relationship with mental health problems. We thus hypothesised that:

*Hypothesis* 4: People who score higher on nationality will report lower levels of corruption perception and weaker relationship between corruption perception and mental health problems.

Similarly, no literature was found to help us formulate the relationship between a person’s place of living (i.e., living in a rural or urban area, or both) and corruption perception, and its effects on mental health. We may draw from the level of exposure to corruption in the rural–urban divide to formulate a hypothesis. Residents in an urban area are expected to have more contact with public administration than residents in rural areas. This means that rural residents will be less likely to be exposed to corruption, and therefore, their levels of corruption perception would be lower as would be the effect of corruption perception on mental health problems. For this, our hypothesis was:

*Hypothesis* 5: People who have lived the most part of their lives in urban areas will report higher levels of corruption perception and stronger relationship between corruption perception and mental health problems.

### A network approach to corruption perception

Different aspects of corruption perception are thought to coexist with each other and mutually influence its occurrence. With this in mind, we employed a novel statistical approach—*psychological network analysis*—to examine the mutual interdependence or associations between four aspects of corruption perception, thus opening a way to empirically determine and provide specific information about how different aspects of corruption mutually interact, often reciprocally, and reinforce each other. With the network approach, it is also possible to examine how multiple constructs interact in a system of complex network. For instance, it is possible to examine how different aspects of corruption perception is related to other attributes in another community such as interpersonal trust or mental health problems through the so-called *bridge connections*. Therefore, it is possible to identify important associations between specific aspects of corruption perception and particular mental health problems. This is also important in our study since we believe that different aspects of corruption are more or less strongly linked differently to interpersonal trust and symptoms of mental health problems. When a person is denied access to critical healthcare because of corruption in state institutions thereby resulting in a fatal outcome, that person may not only worry but spiral into repetitive negative thinking such as rumination with a sense of loss, failure and hopelessness—predominant features of depression (25)—which could mean that this aspect of corruption is probably linked to both anxiety and depression whereas the perception that rich people in society can influence state institutions may only be linked to worrying, uncontrollability and a diminished expectation of personal agency due to inadequate financial resources—predominant features of anxiety (26, 27). Similarly, witnessing a corrupt transaction involving a bribe-seeking state official may not be linked with suicide risks, but could significantly erode trust in state institutions and the governance accountability structures. These nuances may contribute to reconceptualising corruption as a key social determinant of public mental health that may inform targeted therapeutic interventions in mental health practice and intervention science. Due to the exploratory nature of network analysis, we adopted an atheoretical approach and made no specific hypotheses regarding the network analysis.

#### The present study

In the present study, we aimed to expand our understanding of corruption perception among a sample of Ghanaian university students. Specifically, our aims were to (i) assess the overall levels of corruption perception among different social groups, and (ii) assess differences in the effects of corruption perception on mental health problems, (iii) model a network of corruption perception, and finally, (iv) to model a combined network of corruption perception by integrating multiple communities of relevant variables (interpersonal trust, symptoms of anxiety and depression, and suicidal risk as measures of mental health problems). In doing so, we contribute to a nascent literature on the mental health consequences of corruption while controlling for trust in interpersonal relationships. To the best of our knowledge, this is the first study to investigate corruption perception and empirically determine the most central aspects among a constellation of corruption perception indicators in a network analysis. Additionally, it is the first study to determine which aspect of corruption perception strongly links with other relevant variables including interpersonal trust and mental health problems estimated in a combined network.

## Methods

### Participants and procedure

Students at the University of Ghana (UG) and Akenten Appiah-Menka University of Skills Training and Entrepreneurial Development (AAMUSTED) were invited to take part in the study. Four hundred and eighty participants from AAMUSTED (mean age = 23.24 years, *SD* = 3.75 years; 70.20% males) completed a paper-and-pen survey and 250 participants from both UG and AAMUSTED (mean age = 20.01 years, *SD* = 2.33 years; 42.80.20% males) completed an online survey. Overall, there were 730 (*N* = 730; 60.8% men) participants with a mean age of 22.13 years, *SD* 3.66 years. The project was approved by the Regional Committees for Medical and Health Research Ethics in Norway—108,321 and the University of Ghana Ethics Committee for the Humanities—ECH 187/19–20.

### Instruments

#### Corruption perception among state institutions and actors

Participants’ corruption perception was measured using the following four items: *“State institutions are corrupt (*e.g.*, public universities, hospitals),” “Politicians and other government officials are corrupt (*e.g.*, MPs, MMCES, ministers),” “In this country, people who have money/resources can influence any state institution(s) or government official(s) for personal gains,” “In the last 12 months, I have seen a person influence/induce state institution(s) or government official(s) with money or other thing for personal gains.”* Responses ranged from ‘Not at all’ (1) to ‘Extremely likely’ (4).

#### Interpersonal trust

For measuring interpersonal trust, the widely used three items related to trust in people, human fairness and human nature was used (28, 29). Trust in people is measured by, “*Generally speaking*, *would you say that a) most people can be trusted; or do you think b) you cannot be too careful in dealing with people?.”* Trust in human nature is measured by *“Would you say that a) most of the time people try to be helpful; or that b) they are mostly looking out for themselves?.”* Trust in human fairness was measured by asking *“do you think that a) most people would try to be fair; or do you think that b) they would try to take advantage of you if they got the chance*. Participants were required to choose one response option. Choosing the first sentence in the highest agreement was considered to have a higher level of trust, while the second sentence was considered as a lower level of trust.

#### Anxiety and depression symptoms

The Hopkins Symptom Checklist-short form (HSCL-10) (30) is a 10-item self-report questionnaire assessing psychological distress, comprising symptoms of anxiety and depression. Symptoms of anxiety and depression form the two subscales of HSCL. Four of the 10 items assess levels of anxiety while the remaining six items assess levels of depression. Respondents rated each questionnaire item on a scale from ‘Not at all’ (1) to ‘Extremely’ (4).

#### Suicide risk

Three questions form the Suicide Behaviours Questionnaire-Revised (SBQ-R) (31) were selected to assess lifetime risk of suicide scored from “*Never”* to “*I have attempted to kill myself, and really hoped to die.”* Suicide ideation over the past 12 months was scored from “*Never*” to “*Very Often (5 or more times),* and the threat of suicide attempt was scored from “*No*” to “*Yes, more than once, and really wanted to do it*.” The SBQ-R has been validated for use in general population samples (31).

### Statistical analyses

Basic correlation analyses, group mean difference tests (i.e., independent samples *t*-test and analysis of variance) and moderation analyses using multivariate regression models were performed. Mental health problem was computed as the sum score of suicide risk, anxiety, and depression symptoms. Gender, age, religiosity, nationality, and place were entered as moderator variables in separate analyses with corruption perception as independent variable. The network analysis was used to map out the network structure between items measuring the corruption perception. Network components in this study were the questions measuring corruption perception, referred to as nodes. The associations between nodes are referred to as edges (32–34). Statistically, edges represent partial correlations between two nodes, controlling for all the other nodes (33, 34). A correlation matrix of the items was computed and used as input to estimate a Gaussian Graphical Model (GGM) that estimates pairwise association between all indicators (i.e., nodes) (33). The network was estimated using *qgraph* (35), *glasso* (36) and *bootnet* (34) for checking network accuracy and stability. Having computed a network of items measuring the corruption perception, we then computed a combined network comprising items measuring corruption perception, interpersonal trust, and measures of mental health problems (i.e., anxiety and depression symptoms, and suicide risk). The stability of the networks was estimated by calculating the correlation stability (CS) coefficients with the *bootnet* package (34). The CS coefficient should be at least 0.25 and preferably above 0.50 to determine whether the network can be considered stable based on the centrality metrics. The one-step expected influence was calculated to determine which aspect of the corruption perception was most central to the network, representing the relative importance of a node in the network (37). Similarly, the *bridge expected influence (BEI)* was computed to determine which aspect of the corruption perception is most closely related to the other communities of nodes. Thus, we were able to examine which aspect of corruption perception strongly influences, links or is more likely to be a “bridge node” to specific communities while accounting for all relationships in the combined network. All analyses were performed in R version 3.6.3 (38). Additional description of the network analyses is contained in the Supplementary material. The R-scripts are publicly available in the Open Science Framework via the link.^1^

## Results

### Descriptive statistics

Table 1 shows the descriptive statistics for the samples.

**Table 1:** Descriptive statistics.

Variable		N (%)	Corruption perception	Interpersonal trust	Anxiety symptoms	Depression symptoms	Suicide risk
			M(SD)	M(SD)	M(SD)	M(SD)	M(SD)
Gender							
	Female	269 (37.7%)	12.99 (2.85)	0.99 (0.93)	10.88 (3.51)	10.1 (2.91)	4.58 (2.69)
	Male	444 (62.3%)	12.43 (2.95)	1.10 (0.96)	9.64 (3.27)	9.96 (3.13)	3.74 (2.01)
Age							
	Young adults	396 (55.1%)	12.77 (2.87)	0.98 (0.91)	10.24 (3.51)	10.03 (2.95)	4.24 (2.49)
	Old adults	323 (44.9%)	12.38 (3.01)	1.17 (0.99)	9.84 (3.19)	9.98 (3.12)	3.82 (2.08)
Lived mostly							
	Rural and urban	317 (44.4%)	12.37 (2.98)	1.15 (0.98)	9.86 (3.20)	10.29 (3.15)	4.00 (2.21)
	Rural only	149 (20.9%)	12.12 (2.89)	1.22 (0.96)	9.67 (2.99)	9.93 (2.88)	3.65 (2.06)
	Urban only	248 (34.7%)	13.21 (2.80)	0.84 (0.86)	10.51 (3.79)	9.63 (2.97)	4.30 (2.49)
Religiosity							
	Not at all	148 (20.4%)	13.08 (3.27)	0.99 (0.91)	9.96 (3.50)	10.18 (3.10)	4.05 (2.38)
	Moderately	169 (23.3%)	13.25 (2.58)	0.85 (0.94)	10.10 (3.41)	10.04 (2.91)	4.22 (2.14)
	Strongly	243 (33.6%)	12.11 (2.79)	1.10 (0.93)	10.29 (3.36)	9.89 (2.98)	4.21 (2.44)
	Very strongly	164 (22.7%)	12.26 (2.99)	1.30 (0.97)	9.86 (3.41)	10.08 (3.29)	3.68 (2.25)
Nationality							
	Not at all	140 (19.3%)	13.23 (3.10)	1.00 (0.92)	9.83 (3.55)	10.02 (2.79)	3.94 (2.19)
	Moderately	165 (22.7%)	13.04 (2.69)	0.91 (0.89)	10.12 (3.32)	9.88 (3.36)	4.61 (2.82)
	Strongly	212 (29.2%)	12.24 (2.87)	0.99 (0.94)	10.19 (3.43)	9.96 (2.98)	4.20 (2.50)
	Very strongly	210 (28.9%)	12.21 (2.97)	1.30 (0.98)	10.10 (3.38)	10.02 (3.06)	3.57 (1.56)

N, number of participants who responded. Missing data are unreported. M, Mean score. SD, Standard deviation.

### Gender effect

Women reported slightly higher corruption perception than men (*Mean Difference, MD* = 0.56, 95% CI [0.12, 1.00], *t*(569.08) = 2.49, *p* < 0.05; Cohen’s *d* = 0.21, 95% CI [0.04, 0.37]). Women did not report stronger relationship between corruption perception and mental health problems than men. Hypothesis 1 was, therefore, partly supported.

### Age effect

Young and old adults did not differ in their levels of corruption perception, and young adults did not report stronger relationship between corruption perception and mental health problems than old adults. Hypothesis 2 was not supported.

### Sociocultural variables

#### Religiosity effect

**Figure 1:**
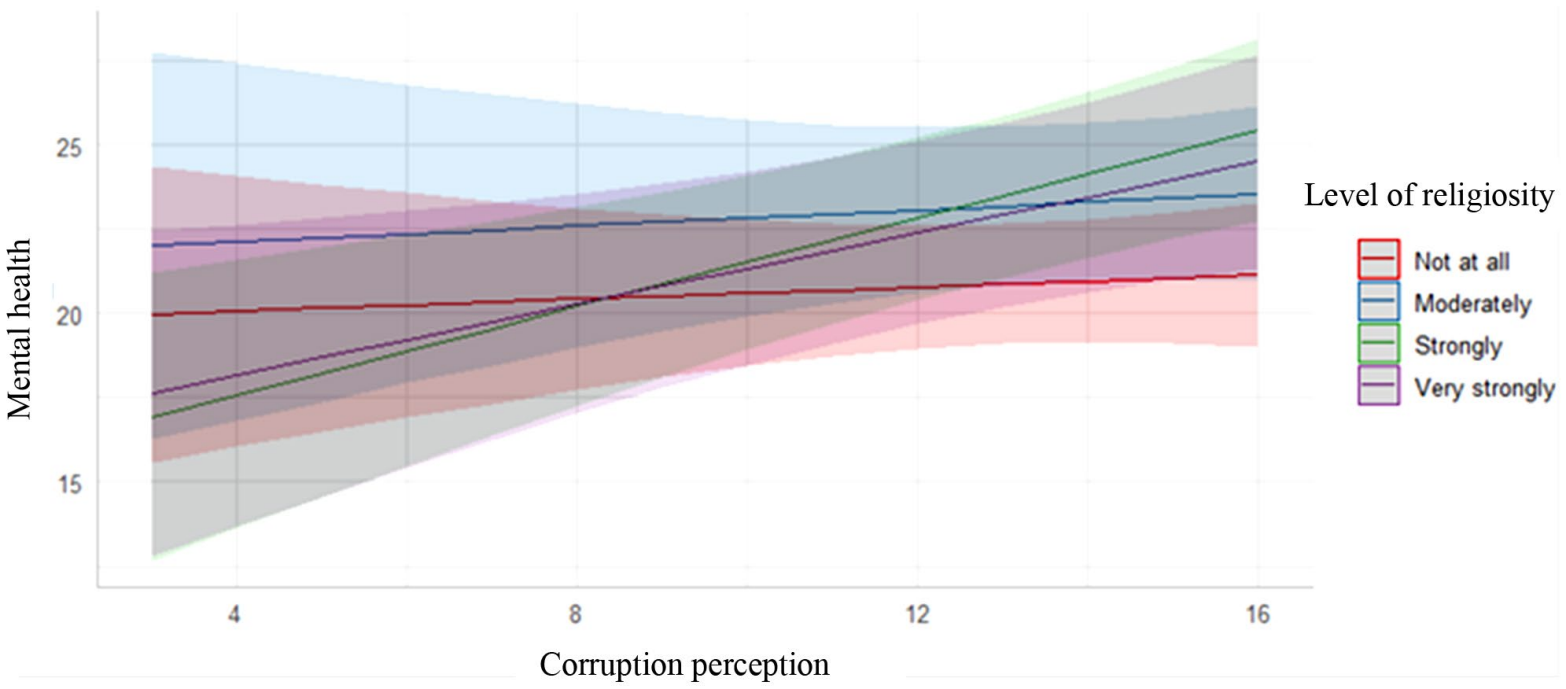
Interaction plot for the relationship between corruption perception and mental health for various levels of religiosity. The slope linking corruption perception to mental health was steeper and significant among those who responded “Strongly”.

There was a significant difference between levels of religiosity on corruption perception, *F*(3, 710) = 7.154, *p <* 0.001, partial *η^2^* = 0.029. Participants who responded “*Moderately*” reported higher corruption perception than those who responded “*Strongly*” (*MD* = 1.14, 95% C.I 0.318, 1.960) and *“Very strongly”* (*MD* = 0.99, 95% C.I 0.097, 1.890), respectively. Those who responded, “*Not at all*” reported higher corruption perception than those who responded “*Strongly*” (*MD* = 0.97, 95% C.I 0.118, 1.817). Other pairwise comparisons were not significantly different. There was a significant interaction between religiosity and corruption perception on mental health problems, controlling for all other variables, *F*(7, 666) = 4.89, *p* < 0.001. The interaction plot showed that the effect of corruption perception on mental health was consistently positive across all the levels of religiosity, but stronger for those who responded “*Strongly*” and “*Very strongly*” to their levels of religiosity (See Figure 1). Hypothesis 3 was, therefore, partly supported.

#### Nationality effect

There was a significant difference between levels of nationality on corruption perception, *F*(3, 712) = 5.682, *p <* 0.001, partial *η^2^* = 0.023. Participants who responded, “*Not at all*” reported significantly higher corruption perception than participants who reported “*Very strongly*” (*MD* = 1.016, 95% C.I 0.122, 1.910), and *“Strongly”* (*MD* = 0.990, 95% C.I 0.099, 1.882). Other pairwise comparisons were not significantly different. No significant group differences were found for the relationship between corruption perception and mental health problems. Hypothesis 4 was, therefore, partly supported.

#### Place effect

There was a significant difference between where people have lived the most in life on corruption perception, *F*(2, 700) = 8.445, *p <* 0.001, partial *η^2^* = 0.023. Those who lived only in urban areas reported significantly higher corruption perceptions than participants who lived in both rural and urban areas (*MD* = 0.84, 95% C.I 0.232, 1.450) or rural areas only (*MD* = 1.097, 95% C.I 0.351, 1.844). Other pairwise comparisons were not significantly different. No significant group differences were found for the relationship between corruption perception and mental health problems. Hypothesis 5 was, therefore, partly supported.

#### Network structure for corruption perception

**Figure 2:**
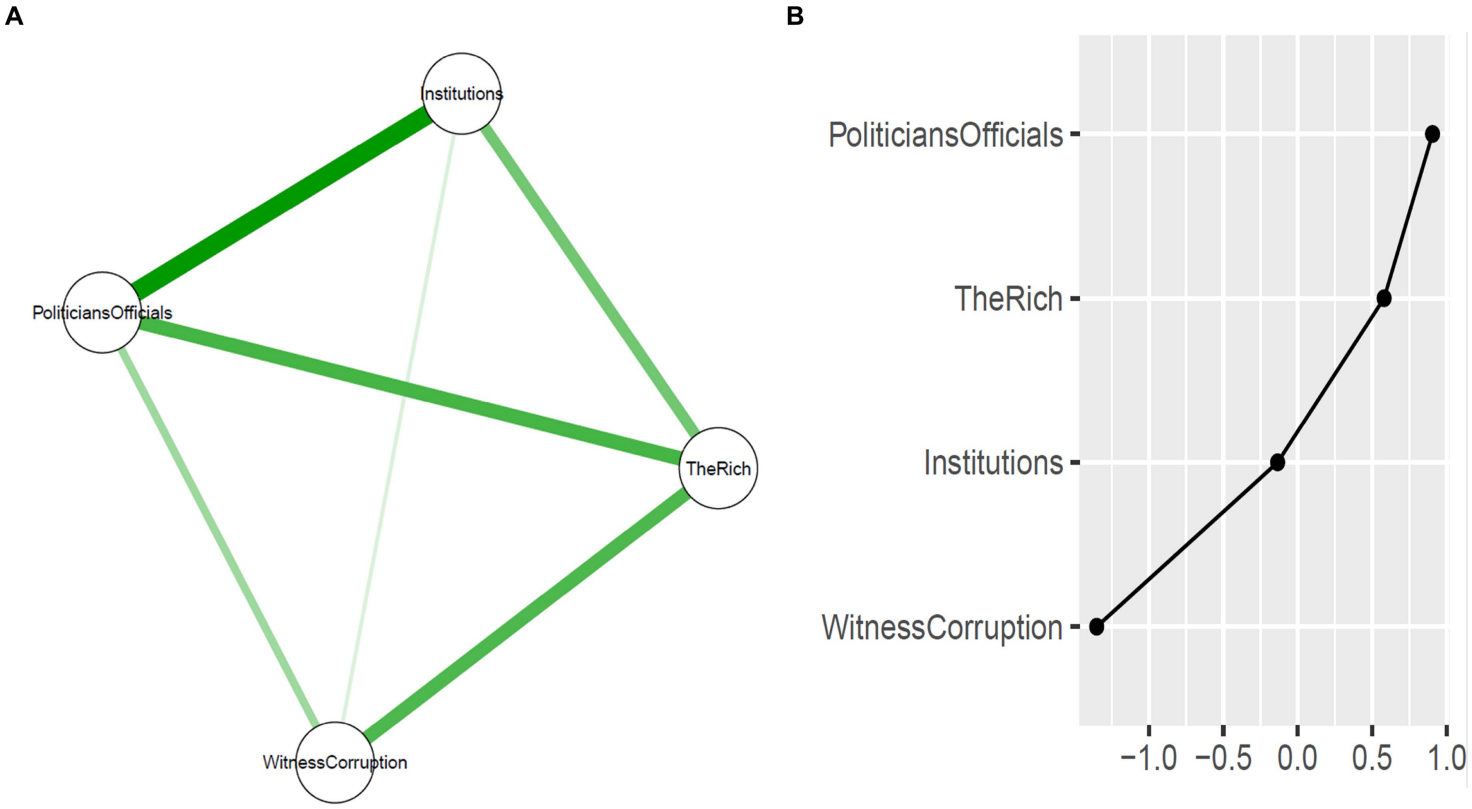
**(A)** Regularised partial correlation network structure containing the four items measuring perception of corruption among state institutions and actors. Green lines represent positive partial correlations while the thickness indicates strength of connection. **(B)** Expected influence metrics for each item. Selected items measuring the perception of corruption are: 1. State institutions are corrupt (e.g., public universities, hospitals). 2. Politicians and other government officials are corrupt (e.g., MPs, MMCES, ministers). 3. In this country, people who have money/resources can influence any state institution(s) or government official(s) for personal gains. 4. In the last 12 months, I have seen a person influence/induce state institution(s) or government official(s) with money or other things for personal gains.

Prior to estimating the combined network of corruption perception, interpersonal trust, and mental health problems, we estimated a network of only corruption perception. Figure 2A shows a visualisation of the network structure of all four items measuring corruption perception. Overall, the items were positively connected within the network, and especially strong connections emerged between the perception that “*state institutions are corrupt*” and the perception that “*politicians and other government officials are corrupt*.” Figure 2B illustrates estimates of the expected influence in the network. The perception that “*politicians and other government officials are corrupt”* has the highest expected influence. This means that, from a statistical point of view, this is the highly connected node in the network with the most influence. The correlation stability (CS) coefficients revealed an acceptable level of stability in the network for edge weights (*CS* = 0.52), expected influence (*CS* = 0.59), and closeness (*CS* = 0.36), especially supporting our reliance on the expected influence (whose CS is above the threshold of 0.25) for interpreting the relative influence of highest or lowest nodes. Supplementary Figures S1A,B display additional results for the item network accuracy and stability analyses as well as standardised estimates for betweenness, closeness and strength centrality indices.

#### Network structure of corruption perception, interpersonal trust, and mental health problems

**Figure 3:**
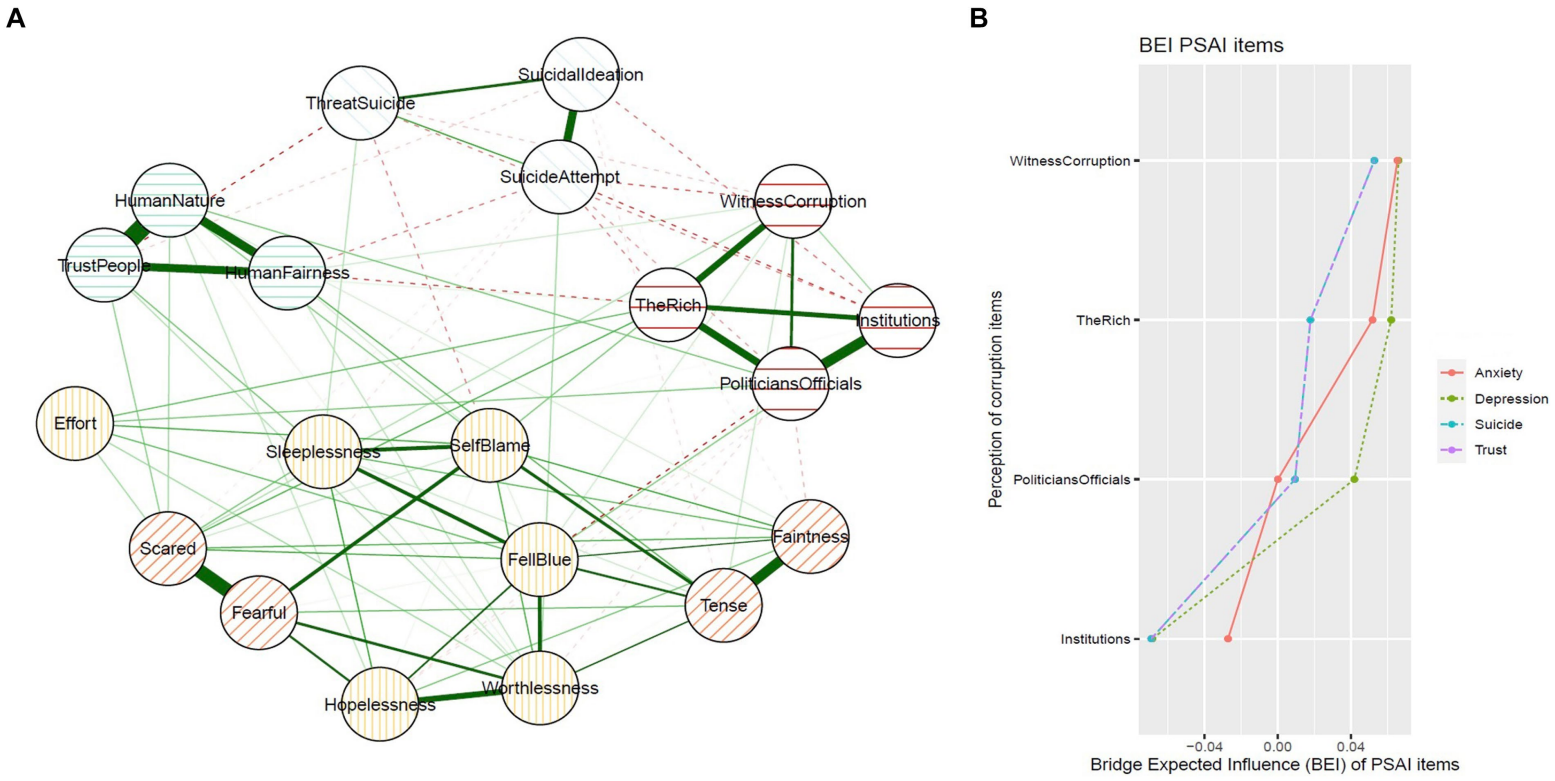
**(A)** Regularised partial correlation network of items measuring perception of corruption, interpersonal trust, suicide risk, symptoms of anxiety and depression. **(B)** Bridge expected influence values for each perception of corruption item with respect to interpersonal trust, suicide risk, symptoms of anxiety and depression. PSAI, Perception of corruption among state institutions and actors. BEI, Bridge expected influence.

A depiction of the combined network structure is contained in Figure 3A. Figure 3B shows the bridge expected influence values of corruption perception items with respect to item communities of interpersonal trust, suicide risk, symptoms of anxiety and depression. Overall, corruption perception items showed much higher bridge expected influence values with respect to the depression community followed by the anxiety community than it did the interpersonal trust and suicidal risk communities. *Witnessing corruption among state institutions and government officials* emerged as the aspect of the corruption perception most likely to bridge corruption perception and depression community as well as the anxiety community. The perception that the “*rich can influence any state institutions or government officials for personal gains”* was the next aspect of corruption perception bridging with depression and anxiety communities, respectively. The correlation stability (CS) coefficients revealed an acceptable level of stability in the combined network for edge weights (*CS* = 0.75), expected influence (*CS* = 0.67), closeness (*CS* = 0.13), and bridge expected influence (*CS* = 0.59). Interpreting the bridge expected influence (whose CS is above the threshold of 0.25) is thus supported.

## Discussion

This study explored corruption perception and its effects on mental health problems. Specifically, the study aimed to (i) assess the overall levels of corruption perception, and (ii) examine differences in the effects of corruption perception on mental health problems. The study also modelled (iii) a network of corruption perception, and (iv) a combined network of corruption perception and integrated multiple communities of relevant variables (interpersonal trust, symptoms of anxiety and depression, and suicide risk as measures of mental health problems).

Consistent with the study by Goel and Nelson (14) and the findings by Transparency International (4, 13), women reported slightly higher levels of corruption perception than men. The finding is also partly consistent with hypothesis 1 since, the effect of corruption perception on mental health problems did not vary between men and women. Perhaps, documented evidence by agencies such as Transparency International (4, 13), suggesting women’s constant exposure to bribery and other forms of corrupt acts in public institutions in sub-Saharan Africa, heightens women’s vulnerability to and perception of corruption. Differences due to power imbalance in the participation of public versus domestic life could also potentially account for differences in the ways women and men perceive corruption differently. The levels of exposure to perpetration of corruption may also influence the differential gender perceptions of corruption. According to Bauhr and Charron (39), factors such as role socialisation, social status and life experiences may make men and women perceive and engage in different kinds of corruption. The authors drew a distinction between ‘need’ and ‘greed’ corruption and suggested that women are more likely than men to perceive that corruption is driven by need rather than greed. Perhaps driven by their greater care responsibilities, women may be more likely to be exposed to need than greed corruption (39). Though our study did not differentiate between need and greed corruption *per se*, we may agree with Bauhr and Charron (39) that role socialisation may likely influence the differential exposure to and perception of corruption between men and women. While perception may or may not reflect the realities, scholars are divided regarding which of the genders predominate in the perpetration of corruption. Whereas Boehm and Sierra (40) reported that men are more likely to pay bribes and thus participate in corruption more than women, Alolo (15) concluded that women may not prove any less corrupt than men when exposed to corruptible public sector environments.

Hypothesis 2 was not supported as no age difference was observed in corruption perception, and also, the effect of corruption perception on mental health problems did not vary between young and older adults. This finding is counter to evidence suggesting that young people are more likely to engage in paying bribes than older people (16), and those that suggest higher age correlate with lower justifiability of corruption (17). It was expected that compared to older adults, young adults would have the proclivity to engage in corruption and thus, score higher on corruption perception. However, this was not the case in our study. One reason could be the propensity for young persons to deny or trivialise corruption due to social desirability effect. Another reason could also be due to the fact that our study did not investigate participation in corruption. The findings by Transparency International (16) included more than 47,000 citizens from 35 countries across Africa in the Global Corruption Barometer (GCB), which may explain the significant findings due to larger variations in their sample size. Our findings also suggested that age does not moderate the relationship between corruption perception and mental health problems, which could suggest that observed effect of corruption perception on mental health problems may be independent of variations in age.

By the constitution, Ghana is a secular state, and yet a highly religious nation with more than 94.8% of its population professing one religion or the other (41). Corruption is overtly proscribed within religious groups and therefore, high religiosity should imply a stronger commitment to promoting virtues and eschewing vices of corruption. Based on this, we expected that those who responded, “*Very strongly*” to *“how strongly do you associate yourself with your religion?”* would also report higher corruption perception and a stronger relationship between corruption perception and mental health problems in a country where corruption is endemic. However, hypothesis 3 was only partly supported as those who responded “*Moderately*” and “*Not at all*” reported higher corruption perception than those who responded, “*Very strongly*” or “*Strongly*.” This seem to be supported by the argument that an individual’s level of religiosity does not say much in concluding about their moral or ethical standpoint (21). However, our results further showed that the slope linking corruption perception to mental health was steeper and significant among those who responded “*Strongly*.” Therefore, we can argue that, although religiosity may not necessarily relate with moral or ethical standpoints *per se*, the effect that perceptions of immoral or ethical conducts such as corruption may have on mental health may still be of concern when considering different levels of religiosity. Similar conclusions that religious people experience stronger effect of corruption on their mental health have been supported (11). The stronger effect of corruption perception on the mental health of religious people may not be strange because religious adherents believe that living an upright life, such as abhorring corrupt practices and deception, is part of the general order of existence. To the extent that religion increases a person’s capacity for self-control and evaluative moral self-sanction (42), religious persons who engage in or witness corruption are more likely to be anxious about the act, anticipate the negative effects of corruption and thus likely to engage in persistent rumination. They may also hold negative views of themselves or experience depressive symptoms because of the belief that their corrupt behaviours transgress a sacred moral code of an unseen supernatural Being or watcher.

We expected that higher levels of nationality will yield a lower score on corruption perception and a weaker relationship between corruption perception and mental health problems. Hypothesis 4 was partly supported as those who responded, “*Very strongly”* and “*Strongly*” to “*how strongly do you associate with your nationality?*” reported the lowest corruption perception, respectively. We may argue that, based on our definition of nationality in this study, those who scored higher on nationality may be cognitively motivated to underreport or conceal the negative perceptions in the country, including the endemic corruption. It may be cognitively conflicting and discomforting for people who are highly committed to and proud of their country (including the readiness to correct the ills of their country) to hold negative perception (such as high corruption perception) about that country. Thus, to reduce this seeming cognitive dissonance or discomfort, and in order to appear patriotic or confirm one’s allegiance to their country, at least in the eyes of an observer, people who score high on nationality are less likely to accurately report about corruption in their country. This finding may probably be due to the fuzziness of the variable nationality as used in this study as well as lack of conceptual precision of the term in the literature. In this study, we construed nationality to mean membership of a country, which makes it difficult to tap into the psychological and emotional dimension of the term. Still, we may speculate that where corruption perception is endemic, people may possibly internalise corruption as a way of life. Thus, habituation of corruption might work to normalise the experiences and perceptions among most people. This may also explain why there was no significant interaction effect between nationality and corruption perception on mental health problems in this study, partly contradicting hypothesis 4.

Participants in this study who have mostly lived in urban areas reported higher corruption perception than participants who have mostly lived in both rural and urban areas or mostly in rural areas only, partly supporting hypothesis 5. It is important to underscore that, perceptions are partly shaped by experiences and therefore, we can speculate that the more people experience corruption, both as victims and as perpetrators, the more likely they will report higher levels as far as corruption perception is concerned. Most state institutions are located in the urban centres, which thus fosters frequent and deeper interactions between state institutions/actors and urban residents. Given that the rural areas of Ghana are known to consist of people with “deeply entrenched traditional norms and values” (42) (p. 6), it is plausible to suggest that the prohibiting or restricting functions of traditional social control measures in the form of taboos and other customs in rural communities in Ghana may dissuade acts of corruption among rural dwellers, with a consequent lower levels of corruption perception. Furthermore, lower population density in the rural areas make acts of corruption easily noticeable in rural settings than in the urban areas. This situation can deter and limit engaging in acts of corruption in the rural settings and hence minimise perception of corruption. We did not find group differences in the relationship between corruption perception and mental health problems, suggesting that the effect of corruption perception on mental health problems may be independent of where people have mostly lived despite the fact that urban dwellers may report higher corruption perception.

The network analyses provided new and interesting information about the mutual interconnections between the different aspects of corruption perception. The perceptions that “*state institutions are corrupt*” and “*politicians and other government officials are corrupt*” formed the strongest connection with the latter showing the highest expected influence. This means that, people’s perception of corruption among politicians and other government officials is highly influential in affecting other aspects of corruption (e.g., corruption among state institutions). Thus, supporting the notion that various aspects of corruption coexist with each other and mutually influence its occurrence. For instance, the private wealth seeking behaviour of corrupt state officials would thrive when the state and public institution themselves are also corrupt. Consequently, people who have much more money and personal connections will be favoured. Those without adequate material resources or personal connections are significantly disadvantaged (43). Afterall, it is only the rich in society who can dare to influence state institutions and actors with the power of wealth. Impliedly, where corruption becomes a way of life of a people, the poor get ‘crowded out’ of corruption enterprise because of their inaccessibility to the ‘means’ for influencing state institutions and actors.

Additionally, corruption perception showed stronger links to the depression symptoms community followed by the anxiety symptoms community as evidenced by its nodes’ higher BEI values, supporting the idea that the effect of corruption perception is stronger for some mental health problems than others, and also stronger than interpersonal trust. Specifically, “*witnessing corruption among state institutions and government officials*” followed by the perception that the “*rich can influence any state institutions or government officials for personal gains*” emerged as the aspects of the corruption perception most likely to bridge the depression community first, followed by the anxiety community. This may be understandable because when people become aware that corruption is a way of life, and that only a select few have what it takes to live this way of life, they may also become helpless and hopeless and may thus experience a sense of diminished personal agency, all of which are related to anxiety and depression symptoms. This finding brings into sharp focus the question of what aspect of corruption exerts the greatest impact and on what kind of mental health problems.

Firstly, witnessing corruption may reinforce the negative beliefs of harassment, uncertainty and arbitrariness that are prominent in corruption, or it may heighten people’s expectations that either themselves or their loved ones could be unfairly involved or targeted for corruption, which may explain the relation with depression and anxiety symptoms. Harassment, uncertainty, and arbitrariness by someone who represent the state or public authority can become a stressor that may elevate the levels of mental health problems such as depression (3, 11). In a largely communitarian context such as Ghana, the links between witnessing corruption and mental health problems could also be traced to a threefold alternative explanations: which is the tendency for people to (i) remain silent (to protect or save others from being caught in order to maintain relationships), (ii) report (to get the person sacked and ruin relationships) or (iii) conform to a culture of corruption (either in solidarity or for fear of being victimised). These tendencies can create some psychological dissonance that can lead to mental health outcomes such as anxiety and depression.

Secondly, financial difficulty and the feeling of being deprived or hindered when competing for the same resources or services may explain a sense of helplessness and diminished personal agency in meeting the financial demand of bribery and corruption, knowing that the rich in society can influence state institutions or government officials for personal gains. According to Van Deurzen (11), corruption by its nature implies that rules regulating access and distribution of social and material resources are dysfunctional. In societies where people can buy their way through, and where public resources are available only to some people through personal connections or money, jobs can be obtained by the rich and their allies, and public goods (e.g., affordable housing) will be allocated to those who have the means to bribe corrupt state officials (11). Those who do not have the means or personal connections may experience a sense of helplessness in meeting the financial demands of corruption, all of which are associated with anxiety and depression (3). Data from the seventh round of the Ghana Living Standards Survey (GLSS7) conducted in 2016/17 showed that far more people are becoming poor. For instance, the report indicates that between 2013 and 2017, 23.4% of the population were defined as poor and 8.2% as extremely poor, occurring predominantly in the rural areas (44). It is, therefore, not surprising that the perception that rich people in society can influence any state institution or government official also most likely bridges with depression and anxiety symptoms even more than interpersonal trust.

There are limitations to this study. First, our networks are estimated from a sample of university students and therefore, the result may not generalise to other samples. This means that our study sample may not be representative of the larger population so future studies should recruit representative samples of the larger population and address samples across the developmental span from adolescents, young and middle adulthood as well as old adulthood to provide more insights into the relationship among age, corruption perception and mental health problems. Future studies could employ a mixed-method approach to triangulate the quantitative results with qualitative interviews to further enhance the robustness of the study and deepen our understanding of the relationship between corruption perception and metal health problems. With a cross-sectional design, causal inferences cannot be drawn, although the network analysis allows for the examination of the importance or centrality of the causal indicators (in this case the items measuring corruption) to be empirically determined (32). It means that, through this approach we can identify important targets of the aspects of corruption for anti-corruption reduction efforts as well as for targeted intervention practice in mental health. However, we cannot interpret whether a given node or aspect of corruption causes or is caused by sharing a connection with another variable. The results should be interpreted only as partial correlation (associations). We did not estimate a domain network structure for corruption perception and the other constructs since our aim was to empirically determine the influence or importance of the indicators measuring the constructs. Future studies of larger samples should address the domain network structure.

## Conclusion

We used cross-sectional data among a sample of university students in Ghana to investigate the overall levels of corruption perception and the moderation of the relationship between corruption perception and mental health problems. In addition, we modelled a network of corruption perception and a combined network of corruption perception by integrating multiple communities of relevant variables (interpersonal trust, suicide risk, and anxiety and depression symptoms). Women reported slightly higher levels of corruption perception than men. No age differences were found. The highest level of corruption perception was reported by those who responded “*Moderately*,” and “*Not at all*” to questions related to religiosity and nationality, respectively. However, the effect of corruption perception on mental health problems was stronger for those who responded “*Strongly*” to the religiosity question. Participants who lived mostly in urban areas reported higher level of corruption perception. Finally, as far as corruption perception is concerned, the aspect that is most influential and starts off other corruption perceptions is the perception that politicians and other government officials are corrupt. The strong links between witnessing corruption among state institutions and government officials and the perception that the rich in society can influence any state institutions and actors, to depression and anxiety symptoms warrant the need to reconceptualise corruption as a key social determinant of public mental health. This will create a synergy between the financial/economic and the mental health systems towards population level interventions for minimising corruption and maximising positive mental health.

## Data availability statement

The raw data supporting the conclusions of this article will be made available by the authors, without undue reservation.

## Ethics statement

The studies involving humans were approved by Regional Committees for Medical and Health Research Ethics in Norway—108,321 and the University of Ghana Ethics Committee for the Humanities—ECH 187/19–20. The studies were conducted in accordance with the local legislation and institutional requirements. The participants provided their written informed consent to participate in this study.

## Author contributions

FA: Conceptualization, Data curation, Formal analysis, Methodology, Project administration, Visualization, Writing – original draft, Writing – review & editing. JA-A: Conceptualization, Data curation, Writing – original draft, Writing – review & editing. SA: Conceptualization, Data curation, Writing – original draft, Writing – review & editing. CA: Conceptualization, Writing – original draft, Writing – review & editing.

## Footnotes

^1^https://osf.io/5mndh
