# Supplementary material for: Mental health problems, interpersonal trust, and socio-cultural correlates of corruption perception in Ghana
Source: Front Public Health. 2024 Feb 28;12:1269579. doi: 10.3389/fpubh.2024.1269579 (PMC10933067; doi:10.3389/fpubh.2024.1269579)
Supplement: Supplementary file 1 [file Data_Sheet_1.docx]

**Supplementary Material**

Mental health problems, interpersonal trust, and socio-cultural correlates of corruption perception in Ghana

Frederick Anyan*^1^, Johnny Andoh-Arthur^2,4^ Stephen Baffour Adjei ^3,4^ Charity Sylvia Akotia^2,4^

^1^Norwegian University of Science and Technology, Trondheim., Norway.

^2^University of Ghana, Accra, Ghana

^3^Akenten Appiah-Menka University of Skills Training and Entrepreneurial Development, Kumasi, Ghana

^4^Center for Suicide and Violence Research, Ghana

^*^Corresponding author: Department of Psychology, Norwegian University of Science and Technology, NO – 7491. Trondheim, Norway.

Email: frederick.anyan@ntnu.no

**Network Analyses of corruption perception**

Different aspects of corruption perception are thought to coexist with each other and mutually influencing its occurrence. For instance, the private wealth seeking behaviour of corrupt state officials would thrive when the state and public intuitions themselves are also corrupt. Consequently, people who have much more money and personal connections will be favoured. With this in mind, we employed a novel statistical approach – *psychological network analysis* – to examine the mutual interdependence or associations between four aspects of corruption. The network analysis was used to map out the network structure between items measuring the corruption perception. Network components in this study were the questions measuring corruption perception referred to as nodes. The causal associations between nodes are referred to as edges (Borsboom & Cramer, 2013; Epskamp, Borsboom, & Fried, 2018; Epskamp & Fried, 2018). Statistically, edges represent partial correlations between two nodes, controlling for all the other nodes (Epskamp et al., 2018; Epskamp & Fried, 2018). A correlation matrix of the items was computed and used as input to estimate a Gaussian Graphical Model (GGM) that estimates pairwise association between all indicators (i.e., nodes) (Epskamp & Fried, 2018). Graphical LASSO (i.e., Least Absolute Shrinkage and Selection Operator) was used to regularize parameters resulting from the GGM to avoid spurious connections. The graphical LASSO algorithm calculates the edges between nodes by computing partial correlations between them, and then applying an L1 penalty to mask off smaller edges. This returns a sparse network consisting of only edges that are likely to be observed above chance. The network was estimated using *qgraph* (Epskamp, Cramer, Waldorp, Schmittmann, & Borsboom, 2012), *glasso* (Friedman, Hastie, & Tibshirani, 2014) and *bootnet* (Epskamp et al., 2018) for checking network accuracy and stability.

***Network Stability analyses:*** The stability of the networks was estimated by calculating the correlation stability (CS) coefficients with the *bootnet* package (Epskamp et al., 2018). A CS coefficient represents the proportion of cases that can be eliminated while keeping a correlation of at least 0.70 with the original centrality metrics of interest within a 95% confidence interval. The CS coefficients were calculated by performing 1,000 case-dropping bootstraps. The CS coefficient should be at least 0.25 and preferably above 0.50 to determine whether the network can be considered stable based on the centrality metrics.

***Expected influence (EI)***: The one-step expected influence was calculated to determine which aspect of the corruption perception was most central to the network. The EI is the sum of a node’s (i.e., items of the questionnaire) connections, representing the relative importance of a node in the network (Robinaugh, Millner, & McNally, 2016). The EI is calculated by summing all edges for a given node, taking the sign of each edge into account. Thus, EI reflects both the strength and direction a given node’s net association with all other nodes, taking into account negative associations (Robinaugh et al., 2016).

***Bridge expected influence (BEI):*** The computation of the BEI is similar to the EI. The BEI considers the edges between a given node and nodes from other communities in its calculation. Having computed a network of items measuring the corruption perception, we then computed a combined network comprising items measuring corruption perception, interpersonal trust, and measures of mental health problems (i.e., anxiety and depression symptoms, and suicide risk) in a regularized partial correlation network using the graphical LASSO algorithm. To determine which aspect of the corruption perception is most closely related to the other communities of nodes namely, interpersonal trust community, symptoms of anxiety, and depression communities and suicide risk community, the BEI estimate for each corruption perception node was used. Thus, we were able to examine which aspect of corruption perception strongly influences or links to specific communities while accounting for all relationships in the combined network. Corruption perception nodes with higher BEI are more likely to be “bridge nodes” that spread activation between corruption perception and either interpersonal trust community, or symptoms of anxiety, and depression communities, or the suicide risk community.


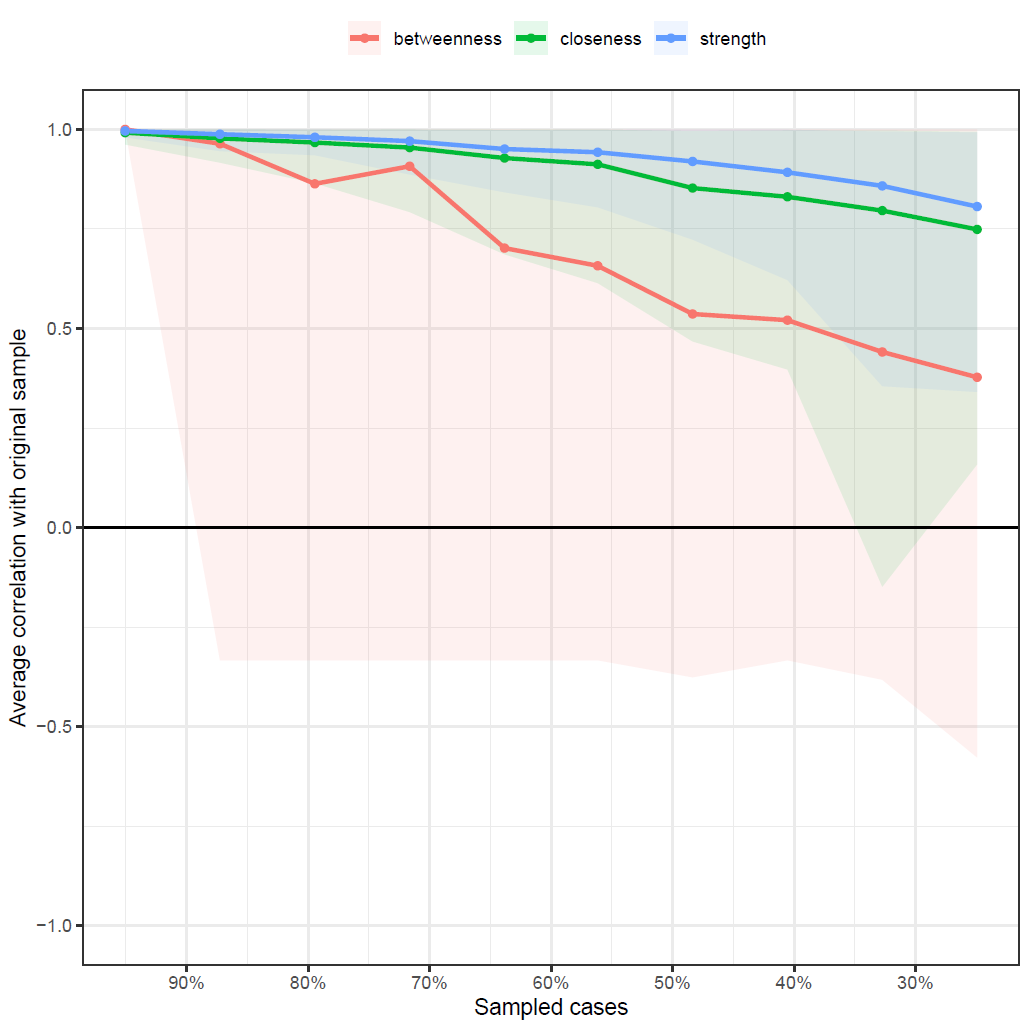


**Fig. S1A.** Stability of centrality indices in the perception of corruption network seem safe for interpretation based on the Correlation stability (CS) Coefficients for strength (0.59) and closeness (0.36). The node strength is highly stable, and thus, interpretations of the relative importance of highest and lowest nodes is safe.


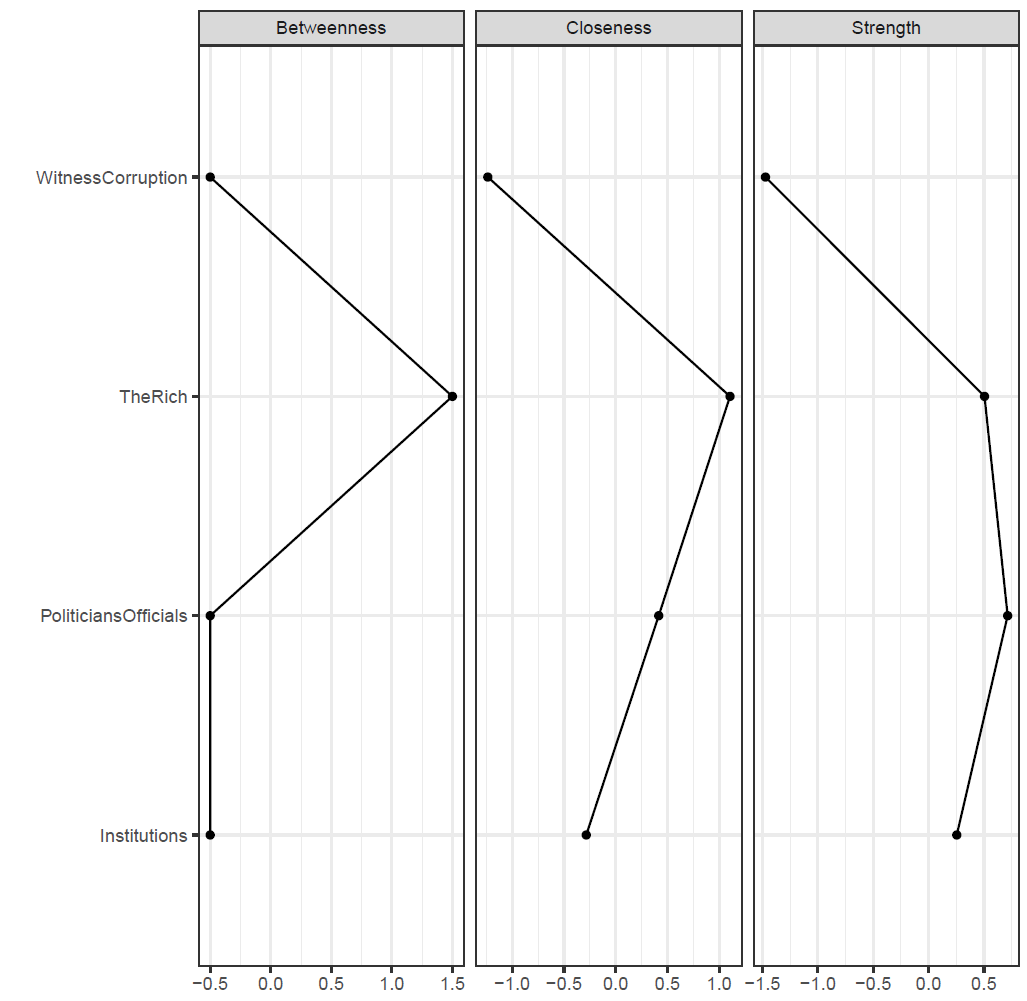


**Fig. S1B.** Betweenness, Closeness and Strength centrality estimates for the perception of corruption network.


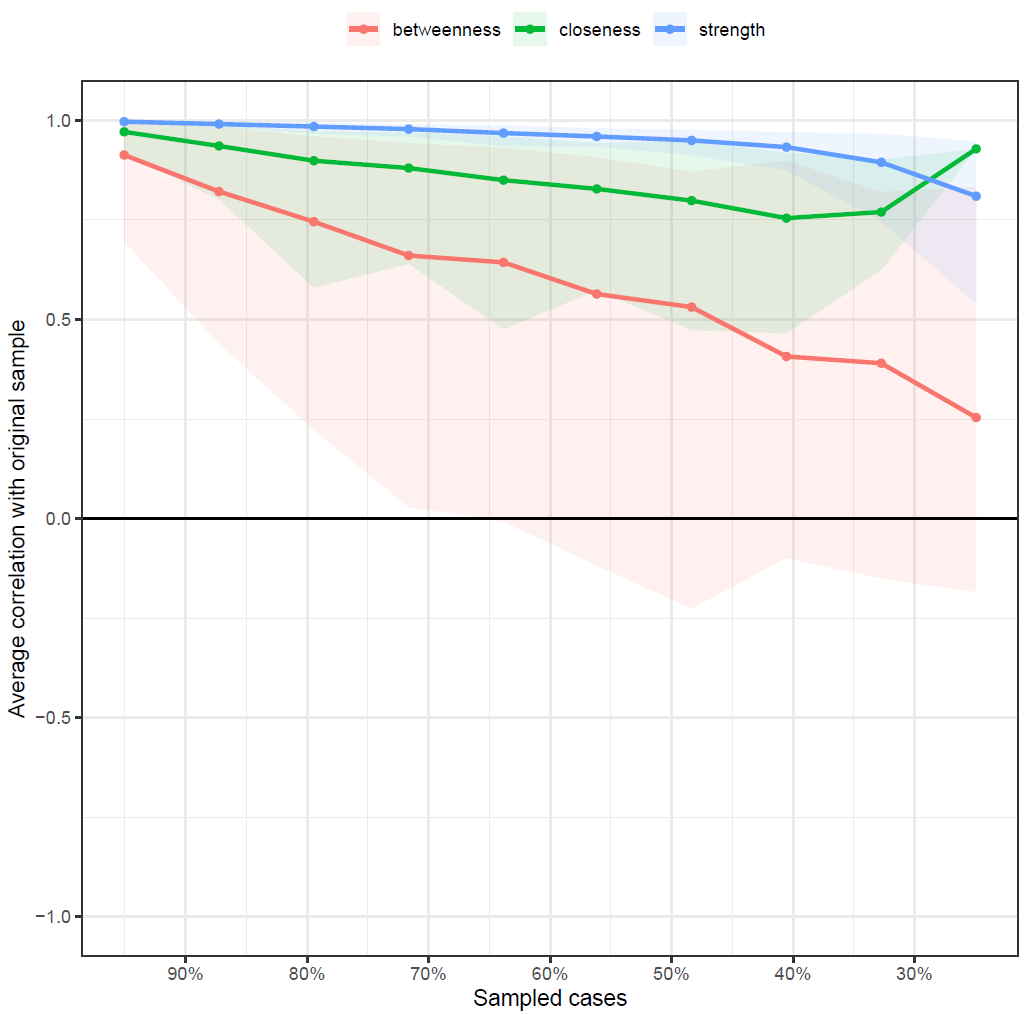


**Fig. S2A.** Stability of centrality indices in the combined network seem safe for interpretation based on the Correlation stability (CS) Coefficients for strength (0.67) and closeness (0.36). The node strength is highly stable, and thus, interpretations of the relative importance of highest and lowest nodes is safe.


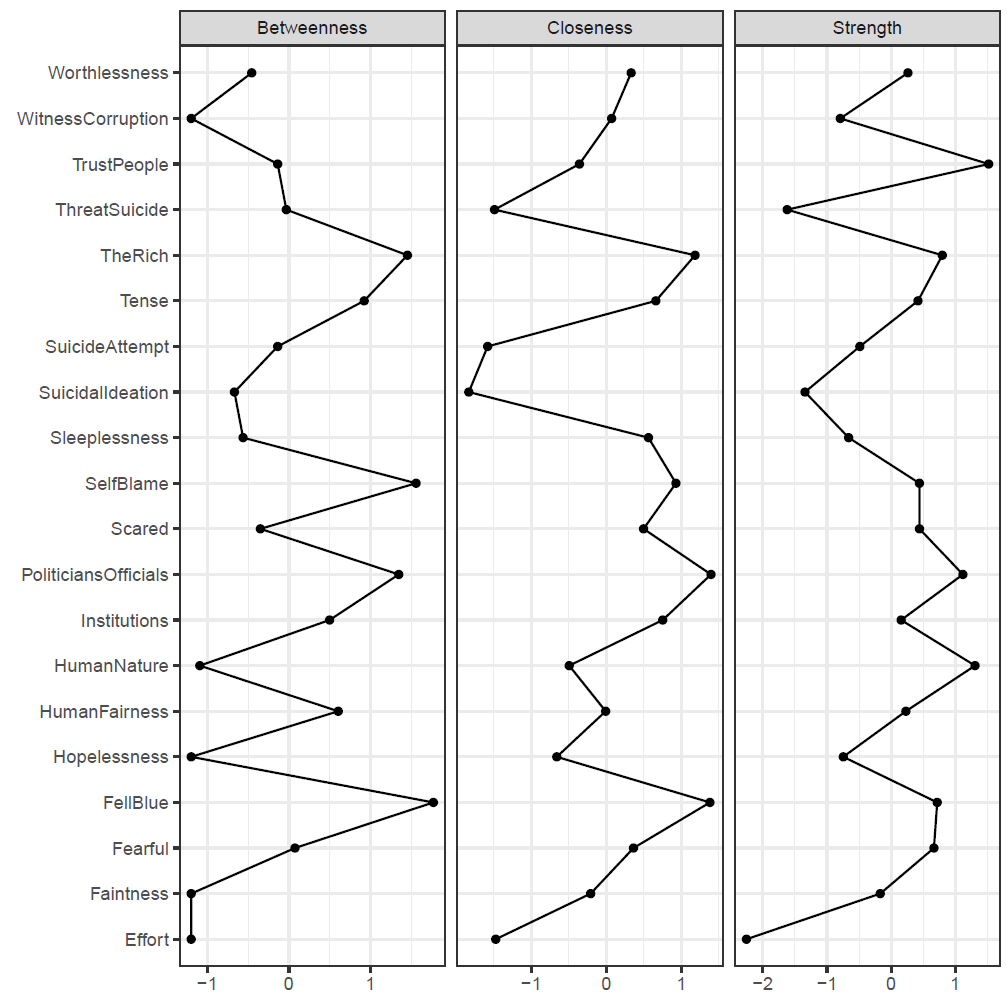


**Fig. S2B.** Betweenness, Closeness and Strength centrality estimates for the combined network structure.

**References**

Borsboom, Denny, and Angélique O. J. Cramer. 2013. “Network analysis: an integrative approach to the structure of psychopathology.” *Annual review of clinical psychology, 9,* 91-121.

Epskamp, Sacha., Denny, Borsboom, and Eiko, Fried. 2018. “Estimating psychological networks and their accuracy: A tutorial paper.” *Behavior Research Methods, 50*(1), 195-212

Epskamp, Sacha., Angélique O. J. Cramer., Lourens J. Waldorp., Verena D. Schmittmann, and Denny Borsboom. 2012. “qgraph: Network visualizations of relationships in psychometric data.” *Journal of statistical software, 48,* 1-18.

Epskamp, Sacha, and Eiko I. Fried. 2018. “A tutorial on regularized partial correlation networks.” *Psychological methods, 23(*4), 617.

Friedman, Jerome., Trevor, Hastie, and Rob Tibshirani. 2014. “glasso: Graphical lasso-estimation of Gaussian graphical models.” *R package version,* 1(8).

Robinaugh, D. J., Millner, A. J., & McNally, R. J. (2016). Identifying highly influential nodes in the complicated grief network. *Journal of abnormal psychology, 125*(6), 747.
